# P2X_3_ receptor upregulation in trigeminal ganglion neurons through TNFα production in macrophages contributes to trigeminal neuropathic pain in rats

**DOI:** 10.1186/s10194-021-01244-4

**Published:** 2021-04-26

**Authors:** Momoko Koizumi, Sayaka Asano, Akihiko Furukawa, Yoshinori Hayashi, Suzuro Hitomi, Ikuko Shibuta, Katsuhiko Hayashi, Fusao Kato, Koichi Iwata, Masamichi Shinoda

**Affiliations:** 1 Department of Dentistry, Jikei University School of Medicine, Tokyo, Japan; 2 Department of Physiology, Nihon University School of Dentistry, 1-8-13 Kandasurugadai Chiyoda-ku, 101-8310 Tokyo, Japan; 3 Department of Oral and Maxillofacial Surgery, Nihon University School of Dentistry, Tokyo, Japan; 4 Department of Neuroscience, Jikei University School of Medicine, Tokyo, Japan; 5 Center for Neuroscience of Pain, Jikei University School of Medicine, Tokyo, Japan

**Keywords:** Trigeminal ganglion, Tumor necrosis factor alpha, Macrophage, P2X_3_, Trigeminal nerve root compression, Trigeminal neuralgia

## Abstract

**Background:**

Trigeminal neuralgia is a characteristic disease that manifests as orofacial phasic or continuous severe pain triggered by innocuous orofacial stimulation; its mechanisms are not fully understood. In this study, we established a new animal model of trigeminal neuralgia and investigated the role of P2X_3_ receptor (P2X_3_R) alteration in the trigeminal ganglion (TG) via tumor necrosis factor alpha (TNFα) signaling in persistent orofacial pain.

**Methods:**

Trigeminal nerve root compression (TNC) was performed in male Sprague-Dawley rats. Changes in the mechanical sensitivity of whisker pad skin, amount of TNFα in the TG, and number of P2X_3_R and TNF receptor-2 (TNFR2)-positive TG neurons were assessed following TNC. The effects of TNFR2 antagonism in TG and subcutaneous P2X_3_R antagonism on mechanical hypersensitivity following TNC were examined.

**Results:**

TNC induced unilateral continuous orofacial mechanical allodynia, which was depressed by carbamazepine. The accumulation of macrophages showing amoeboid-like morphological changes and expression of TNFα in the TG was remarkably increased following TNC treatment. The number of P2X_3_R- and TNFR2-positive TG neurons innervating the orofacial skin was significantly increased following TNC. TNFα was released from activated macrophages that occurred in the TG following TNC, and TNFR2 antagonism in the TG significantly diminished the TNC-induced increase in P2X_3_R-immunoreactive TG neurons. Moreover, subcutaneous P2X_3_R antagonism in the whisker pad skin significantly depressed TNC-induced mechanical allodynia.

**Conclusions:**

Therefore, it can be concluded that the signaling of TNFα released from activated macrophages in the TG induces the upregulation of P2X_3_R expression in TG neurons innervating the orofacial region, resulting in orofacial mechanical allodynia following TNC.

## Background

Many current studies have evaluated the mechanism underlying trigeminal neuropathic pain using animal models of trigeminal nerve injury [1–3]. For example, chronic constriction injury or partial ligation of the infraorbital nerve is known to cause mechanical allodynia in the whisker pad skin [3, 4]. Various molecules such as nitric oxide or neuropeptides are also generated in the injured trigeminal ganglion (TG) neurons and released from TG neurons, resulting in the sensitization of TG neurons [5, 6]. These mechanisms are believed to be involved in orofacial neuropathic pain associated with trigeminal nerve injury.

Alternatively, it is well known that the trigeminal nerve root compression (TNC) by a cerebellar artery is clinically implicated as a cause of trigeminal neuralgia (TN) [7]. The widely used trigeminal neuropathic pain model induced by injury to the terminal branches of the trigeminal nerve is not a perfect model to mimic TN, but sheds light on the mechanisms underlying TN. To clarify the detailed mechanisms underlying TN, a novel animal model of TNC-mimicked TN needs to be developed. It has been reported that p38 phosphorylation in microglial cells is involved in TN using the TNC model developed by dental agar injection in the trigeminal nerve root [8, 9]. However, many questions regarding the mechanisms underlying TN remain unclear.

Activated macrophages are known to produce various molecules such as tumor necrosis factor alpha (TNFα), and TNFα released from macrophages causes direct modulation of neuronal excitability [2]. TNFα is also known to increase in the cerebrospinal fluid and blood of patients with chronic neuropathic pain [10, 11]. In animal experiments, TNFα upregulation in the dorsal root ganglion (DRG) contributes to mechanical allodynia following spinal nerve ligation [12, 13]. Inferior alveolar nerve injury induces the infiltration of macrophages from which TNFα is released in the TG, which appears to be involved in the mechanical hypersensitivity in the whisker pad skin [2].

The ATP-gated P2X_3_ receptor (P2X_3_R), which is expressed in nociceptive primary afferents, plays a significant role in facilitating transduction of noxious mechanical stimuli to the central nervous system [14]. Painful diabetic neuropathy is caused by membrane P2X_3_R upregulation in dorsal root ganglion neurons via protein kinase C activation in rats [15]. Mechanical allodynia following nerve injury caused by experimental lumbar disc herniation is elicited by neuronal hyperactivity in the DRG, attributable to the enhancement of P2X_3_R expression and function [16]. Nevertheless, the mechanisms responsible for the enhancement of membrane P2X_3_R expression and function in sensory neurons following peripheral nerve injury are not well understood. Together, TNFα, which is elevated in the sensory ganglion, may be listed as one of the likely candidate molecules to regulate membrane P2X_3_R expression and function in sensory neurons in neuropathic pain conditions.

In this study, we hypothesized that (1) TNC induces orofacial mechanical hyperalgesia, (2) TNC induces the infiltration of macrophages that release TNFα into the TG, and (3) TNFα signaling in the TG contributes to the increase in P2X_3_R expressing TG neurons and orofacial mechanical allodynia following TNC. To develop an effective therapy for TN, it is critical to establish an appropriate animal model and to elucidate the mechanisms of orofacial mechanical hypersensitivity using this model.

## Methods

### Animals

All experiments in this study were performed using male Sprague-Dawley rats (n = 125, 160–180 g, Japan SLC, Shizuoka, Japan). Transparent polycarbonate cages (length: 48 cm; width: 26.5 cm; height: 21 cm) with bedding made with paper shavings individually housed all rats. The temperature of the breeding facility was controlled at a constant temperature (23°C) and light-dark cycle (light on at 7:00, off at 19:00). All rats were allowed free access to food and water in their cages. All procedures in this study adhered to the National Institutes of Health Guide for the Care and Use of Laboratory Animals and the guidelines of the International Association for the Study of Pain [17]. The Animal Experimentation Committee at Nihon University approved all procedures (AP18DEN005).

### Trigeminal nerve root compression

Rats were anesthetized with intraperitoneal (i.p.) butorphanol (2.5 mg/kg, Meiji Seika Pharma, Tokyo, Japan), medetomidine (0.375 mg/kg, Zenoaq, Koriyama, Japan), and midazolam (2.0 mg/kg, Sandoz, Tokyo, Japan), and TNC was conducted. Briefly, a drill hole (diameter: 1 mm) in the skull right above the location of the left trigeminal root was made after mounting the rat onto a stereotaxic frame. The glass rod that was passed through the drill hole depressed the left trigeminal root (11 mm below the skull surface, 1.7 mm anterior from the posterior fontanelle, 2.8 mm lateral to the sagittal suture). The rod was anchored to the skull bone using three stainless steel screws with dental cement. As a sham control, the glass-rod was anchored to the skull bone without the TNC (7 mm below the skull surface, 1.7 mm anterior from the posterior fontanelle, 2.8 mm lateral to the sagittal suture). The dissected scalp wound was stitched together using a 6 − 0 silk suture.

### Assessment of the whisker pad skin mechanical sensitivity

Rats were seasoned to keep their snout protruding from a cage that was equipped with a small fenestration previously for 5 to 7 days preceding the assessment of the mechanical sensitivity [1]. Their snout could freely withdraw following mechanical stimuli (intensity: 1, 2, 4, 6, 8, 10, 15, 26, 35, 50, or 60 g) by von Frey filaments (Touch-Test Sensory Evaluator, North Coast Medical, Morgan Hill, CA, USA) to the whisker pad skin. After adaptation to the mechanical stimuli under the condition, TNC or sham treatment was performed. Mechanical stimuli were applied to the whisker pad skin in ascending order of the stimulus intensity, and the mechanical head-withdrawal threshold (MHWT) was determined as the lowest intensity that evoked snout withdrawing more than three out of five stimuli (duration: 1 s). A cutoff of 60 g was used to prevent tissue damage. The MHWT was measured under the same conditions every other day for 21 days and was conducted under blinded conditions.

### Immunohistochemistry

A retrograde labeling tracer (7 µL dissolved in saline, FluoroGold (FG); 4 % hydroxystilbamidine) (Fluorochrome, Denver, CO, USA) was injected into the ipsilateral whisker pad skin using a 30-gauge needle on day 4 after TNC or sham treatment, to label TG neurons innervating the ipsilateral whisker pad skin under deep anesthesia with i.p. administration of butorphanol (2.5 mg/kg), medetomidine (0.375 mg/kg), and midazolam (2.0 mg/kg). On day 7 after TNC or sham treatment, rats were perfused transcardially with 4 % paraformaldehyde in 0.1 M phosphate buffer (PB; pH 7.4), following saline perfusion under deep anesthesia with the foregoing solution. The removed TG and depressed trigeminal root were immersed in 4 % paraformaldehyde for more than 3 h at 4 °C, and afterward in 20 % sucrose dissolved in 0.01 M phosphate buffered saline (PBS) for cryoprotection for 12 h. Subsequently, the removed TG and the depressed trigeminal root, which were embedded in Tissue-Tek (Sakura Finetechnical, Tokyo, Japan) were cut in the horizontal plane parallel to the long axis (14 μm). Every 10th section  (7 sections per rat) was thaw-mounted and dried overnight for 12 h at room temperature on a MAS-GP slide glass (Matsunami, Osaka, Japan). Following rinsing with 0.01 M PBS, the sections were reacted with primary antibodies (anti-ionized calcium binding adapter protein 1 (Iba1) rabbit antibody (1:250; Wako, Cat#019-19741, RRID:AB_839504, Osaka, Japan), anti-TNFα goat antibody (1:100; R&D systems, Cat#AF-510-NA, RRID:AB_354511, Minneapolis, MN, USA), anti-tumor necrosis factor receptor-2 (TNFR2) mouse antibody (1:50; Cat#ab8161, RRID:AB_443597, Abcam, Cambridge, MA, USA), and/or anti-P2X_3_ rabbit antibody (1:1000; Cat#RA10109, RRID: AB_2157931, Neuromics, Edina, MN, USA) ) in 0.3 % Triton-X 100 (Merck, Darmstadt, Germany) diluted in 0.01 M PBS with 4 % normal donkey serum or normal goat serum for 12 h at 4 °C. After rinsing, the sections were reacted with secondary antibodies (anti-goat IgG conjugated Alexa Fluor 568 (1:200; Cat#A-11,057, RRID:AB_2534104, Thermo Fisher Scientific, Waltham, MA, USA), anti-mouse IgG conjugated Alexa Fluor 568 (1:200; Cat#A10037, RRID:AB_2534013, Thermo Fisher Scientific), anti-rabbit IgG conjugated Alexa Fluor 488 (1:200; Cat#A27034, RRID:AB_2536097, Thermo Fisher Scientific), and/or anti-mouse IgG conjugated Alexa Fluor 488 (1:200; Cat#A28175, RRID:AB_2536161, Thermo Fisher Scientific) diluted in 0.01 M PBS at room temperature for 2 h. Sections of trigeminal root were reacted with anti-Iba1 rabbit antibody (1:250; Wako) in 0.3 % Triton-X 100 (Merck) diluted in 0.01 M PBS with 4 % normal goat serum for 12 h at 4 °C. After rinsing, the sections were incubated with anti-rabbit IgG conjugated Alexa Fluor 488 (1:200; Thermo Fisher Scientific) diluted in 0.01 M PBS at room temperature for 2 h. After the sections were coverslipped in PermaFluor (Thermo Fisher Scientific), the Iba1-immunoreactive (IR) cells in the trigeminal root and the Iba1-IR and TNFα-IR cells, and FG-labeled P2X_3_-IR TG neurons were counted using a BZ-9000 system (Keyence, Osaka, Japan). Immunofluorescence, which is more than twice as large as the background level, was defined as IR and labeled. There was no IR using the same procedure in the absence of the primary antibody. All immunohistochemical analyses were performed under blinded conditions.

### Western blotting

On day 7 after TNC or sham treatment, transcardial perfusion using saline was performed under the above-described anesthesia. Ipsilateral immediate-removed TG was homogenized in lysis buffer (137 mM NaCl; 20mM Tris-HCl, pH 8.0; 1 % NP40; 10 % glycerol; 1mM phenylmethylsulfonyl fluoride; 10 µg/mL aprotinin; 1 g/mL leupeptin; 0.05 mM sodium vanadate) at 4°C. The homogenized TG was centrifuged at 15,000 rpm for 10 min, and the protein concentration in the supernatant was analyzed using a protein assay kit (Bio-Rad, Hercules, CA, USA) at 4 °C. Each protein sample (30 µg) obtained from the heat-denatured supernatants was subjected to sodium dodecyl sulfate-polyacrylamide gel electrophoresis using a 10 % acrylamide gel (Bio-Rad). Following the transfer of the protein samples to a polyvinylidene difluoride membrane (Trans-Blot Turbo Transfer Pack, Bio-Rad) using a Trans-Blot Turbo (Bio-Rad), the membrane was incubated in 3 % bovine serum albumin (BSA; Bovogen, Essendon, Australia). Afterwards, the membrane was incubated with rabbit anti-TNFα polyclonal antibody (1:200 diluted in Tris-buffered saline mixed with 0.1 % Tween 20 and 3 % BSA, Cat#ab66711, Abcam) for 12 h at 4 °C. Next, the membrane was incubated with donkey horseradish peroxidase-conjugated anti-rabbit antibody (Cell Signaling, Danvers, MA, USA) for 2 h at 25 °C. The band intensity was examined using a ChemiDoc MP system (Bio-Rad) after the detection of antibody binding by Western Lightning ELC Pro (PerkinElmer, Waltham, MA, USA). The examined band intensity was normalized to the β-actin band intensity re-probed with anti-β-actin antibody (1:200; Santa Cruz, Santa Cruz, CA, USA) following bound protein removal by a stripping agent (Thermo Scientific).

### Effect of intra-TG etanercept administration on mechanical sensitivity

Under the foregoing i.p. anesthesia, a drill hole (diameter:1 mm) was bored in the skull immediately above the ipsilateral TG (2.8 mm anterior from the posterior fontanelle, 2.7 mm lateral to the sagittal suture) following skull stabilization onto the stereotaxic frame like the preceding TNC treatment. A guide cannula that was passed through the drill hole compressed the ipsilateral TG (9 mm below the skull surface). Subsequently, the cannula was anchored to the skull bone using dental cement, together with three screws implanted in the skull bone. To confirm the tip of the guide cannula positioning, multi-unit activities were induced by mechanical stimulation of the whisker pad skin using an electrode inserted into the guide cannula. On day 4 after the guide cannula installation, the TNC procedure was conducted as described above. Five microliters of etanercept (1 µg; Pfizer, New York, NY, USA) dissolved in saline or vehicle (saline) was administrated into the TG through the guide cannula under light anesthesia using 2 % isoflurane on day 4 after TNC or sham treatment. The measurement of whisker pad skin MHWT was performed before TNC or sham treatment (Pre), and on days 3, 7, 9, 11, and 18 after TNC or sham treatment.

### Effect of Carbamazepine and A-317491 administration on mechanical sensitivity

I.p. administration of Carbamazepine (CBZ, 50 mg/kg dissolved in saline, Merck) or vehicle (saline) was performed on day 7 after the TNC procedure under light anesthesia with 2 % isoflurane. The measurement of whisker pad skin MHWT was performed at 0, 30, 60, 90, 120, and 180 min after i.p. administration. Next, 5 µL of vehicle or the selective P2X_3_/P2X_2/3_ receptor antagonist, A-317491 (60 µg dissolved in saline, 5-([(3-phenoxybenzyl)[(1 S)-1,2,3,4-tetrahydro-1-naphthalenyl][amino]carbonyl)-1,2,4-benzene-tricarboxylic acid, Merck) was administered subcutaneously into the whisker pad skin under light anesthesia with 2 % isoflurane on day 4 after the TNC procedure. Five microliters of the selective P2X receptor antagonist, TNP-ATP (50 µg dissolved in saline, 2,3-O-(2,4,6-Trinitrophenyl) adenosine 5-triphosphate tetrasodium salt, Merck) was also administered into the whisker pad skin under light anesthesia with 2 % isoflurane, on day 4 after the sham procedure. The measurement of whisker pad skin MHWT was performed at 0, 30, 45, 60, 90, and 120 min after administration.

### Statistical analysis

For the analysis of mechanical sensitivity in the whisker pad skin, nonparametric procedures were selected because the normality and homogeneity of variances was not fulfilled. Data are presented as median and interquartile range (25–75 %). The upper and lower whiskers represent the maximum and minimum values, respectively. The Mann-Whitney or Kruskal-Wallis test followed by Dunn’s multiple comparisons were used as non-parametric procedures. For the analysis of immunohistochemistry and Western blotting, Student’s *t*-test or one-way analysis of variance with Tukey’s multiple comparison test used. Data are expressed as the mean ± standard error. All statistical analyses were performed using GraphPad Prism ver. 8 (GraphPad Prism Software Inc., San Diego, CA, USA). N represents the number of rats that were tested. Statistical significance was defined as *p* < 0.05.

## Results

### Change in mechanical sensitivity after TNC

**Fig. 1:**
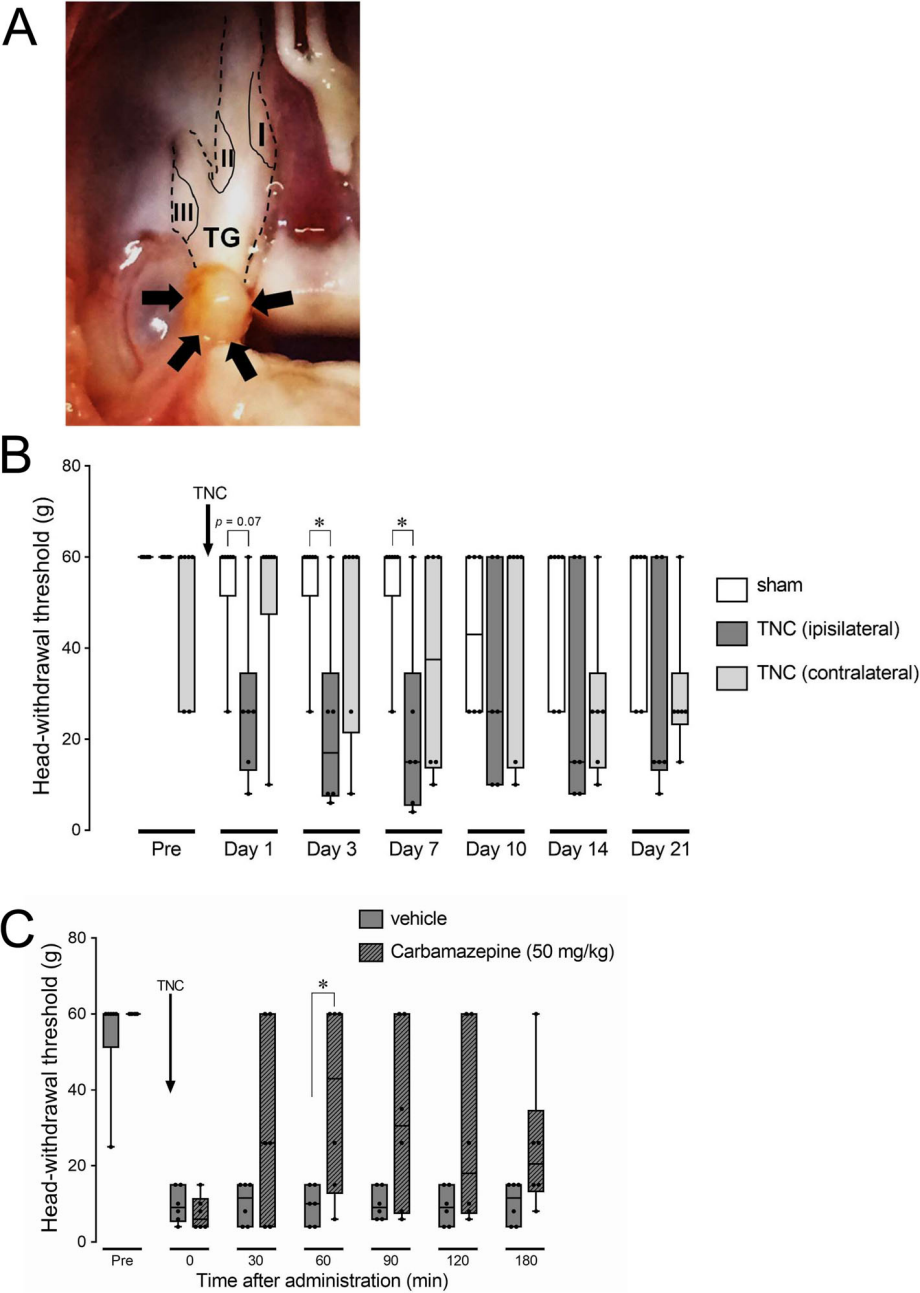
Orofacial mechanical hypersensitivity following TNC and its inhibition by carbamazepine. **a** The trigeminal root following TNC. Arrows indicate the depressed region. I, II, and III indicate the location of neuronal soma innervating to the ophthalmic division, maxillary division, and the mandibular division, respectively. **b** The alteration of whisker pad skin MHWT bilateral to the TNC or sham procedure for 21 days. Data are presented as median, interquartile range (25–75 %), and maximum and minimum values. * *p* < 0.05 vs. sham. (*n* = 6 in each; Kruskal-Wallis followed by Dunn’s multiple comparisons test). **c** The suppressive effect of i.p carbamazepine administration on orofacial mechanical hypersensitivity on day 7 following TNC. Data are presented as median, interquartile range (25–75 %), and maximum and minimum values. * *p* < 0.05 vs. vehicle. (*n* = 6 in each; Kruskal-Wallis followed by Dunn’s multiple comparisons test)

There was no significant difference in MHWT of the bilateral whisker pad skin between the TNC and sham groups before the TNC and sham treatments. TNC induced a significant reduction in MHWT of the whisker pad skin ipsilateral to TNC compared to sham, and in addition contralateral to TNC from day 3 to day 7 (median on day 7, sham: 60 g; TNC (ipsilateral): 15 g, TNC (contralateral): 37.5 g) (Fig. 1a and b). The reduction in MHWT of the whisker pad skin ipsilateral to TNC was recovered from day 10 onwards, and there was no significant difference in MHWT of the bilateral whisker pad skin between the TNC and sham groups on days 10, 14, and 21. The MHWT reduction of the whisker pad skin was significantly suppressed at 60 min after i.p. administration of carbamazepine on day 7 after TNC (median, vehicle: 10 g; carbamazepine: 43 g) (Fig. 1c). No behavioral signs of orofacial spontaneous pain were observed during the experimental period (data not shown).

### Iba1 and TNFα expression in the depressed trigeminal root and TG

**Fig. 2:**
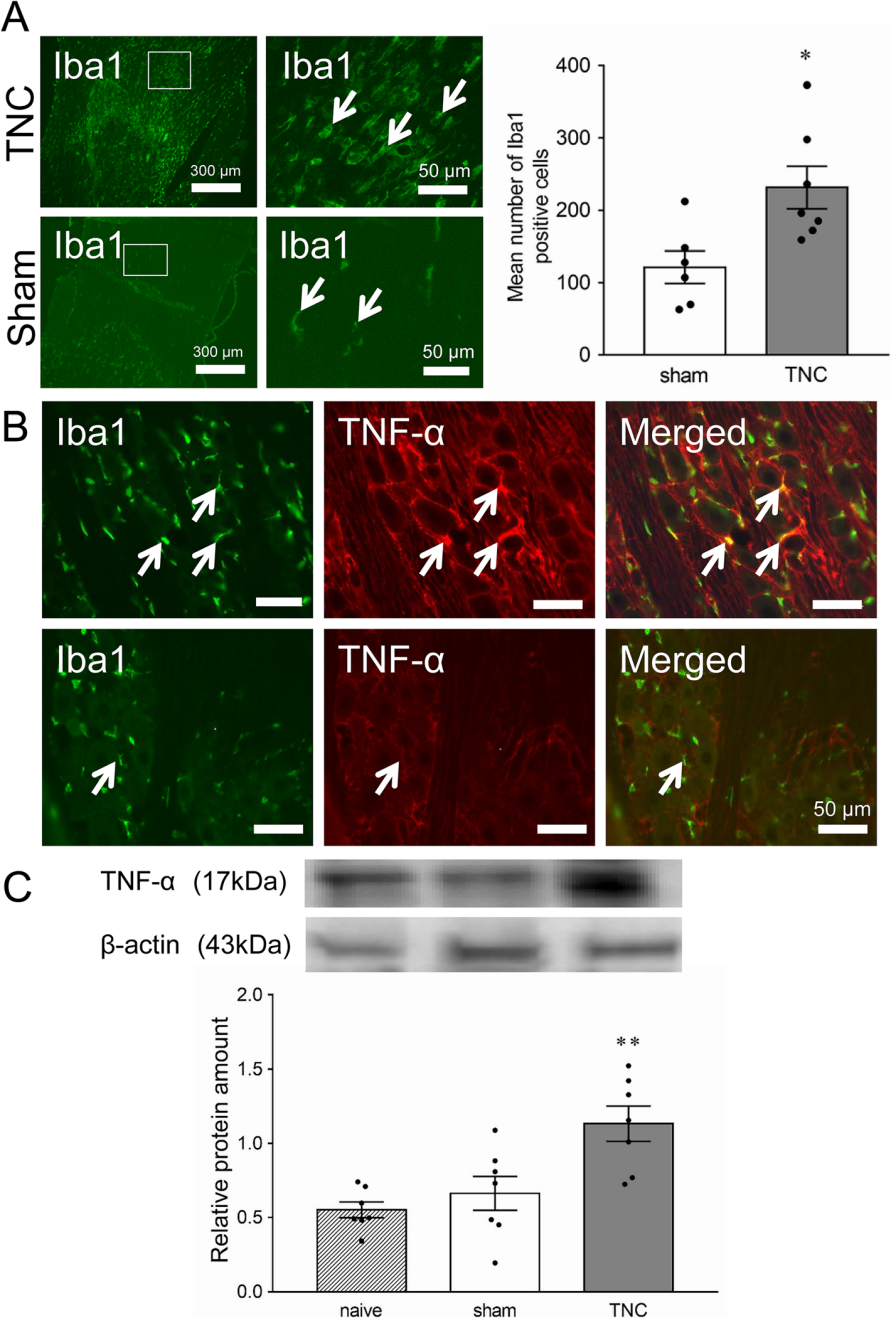
TNFα and Iba1 expression in the TG on day 7 following TNC or sham procedure. **a** Photomicrographs of Iba1-IR cells in the depressed trigeminal root on day 7 following TNC or sham treatment. The right image shows an enlarged image surrounded by a white frame. Arrows indicate Iba1-IR cells in the depressed trigeminal root. Mean number of Iba1-positive cells in the TG section on day 7 following the TNC or sham procedure. * *p* < 0.05 vs. sham. (*n* = 6 in each, student’s *t*-test). **b** Photomicrographs of TNFα-IR and Iba1-IR cells in the TG on day 7 following TNC or sham treatment. Arrows indicate TNFα-IR and Iba1-IR cells in the TG. Scale bar: 100 μm. **c** Relative amount of TNFα protein in the TG in naive rats or on day 7 following the TNC or sham procedure. ** *p* < 0.01 vs. sham. (*n* = 6 in each, F = 9.57, one-way ANOVA with Tukey’s multiple comparison test)

Iba1-IR cells were detected in the compressed trigeminal root on day 7 after TNC, and the mean number of Iba1-IR cells in the ipsilateral TG was significantly greater in the TNC (231.3 ± 31.9, *n* = 7) than in the sham group (121.3 ± 24.7, *n* = 6) (*p* < 0.05, Fig. 2a). Iba1-IR cells were also detected in the ipsilateral TG on day 7 after TNC or sham treatment, and many of them were also TNFα-IR (Fig. 2b). A small number of Iba1-IR and TNFα-IR cells was observed in sham-treated rats. TNFα protein level in ipsilateral TG was significantly higher on day 7 after TNC compared to that of sham or naive animals (naive: 0.55 ± 0.05; sham: 0.66 ± 0.11; TNC: 1.13 ± 0.12, *n* = 7 in each) (*p* < 0.01, Fig. 2c).

### Effect of TNFα disruption on TRNR2 expression

**Fig. 3:**
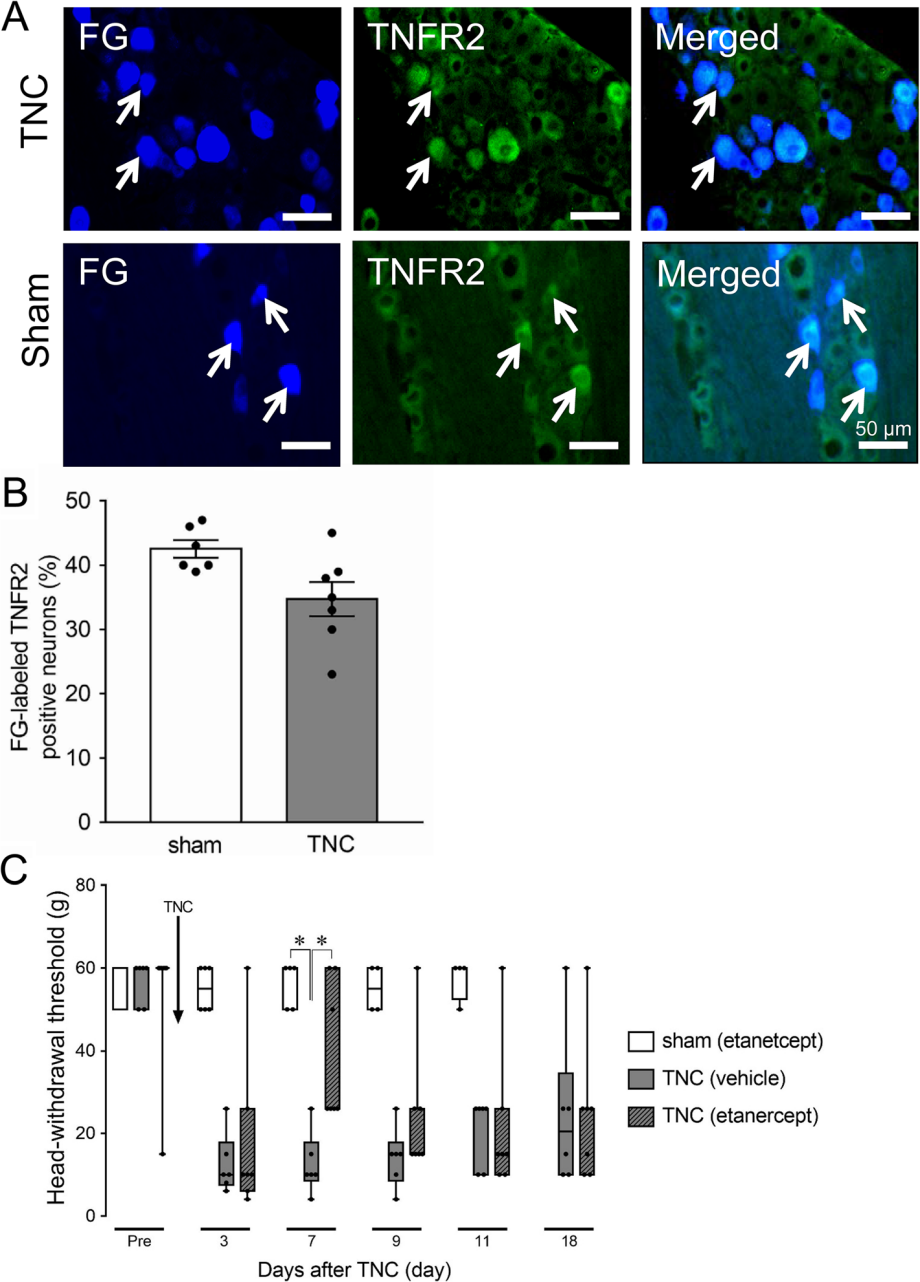
TNFR2 expression in TG neurons innervating the whisker pad skin on day 7 following TNC or sham treatment. **a** Photomicrographs of FG-labeled TNFR2-IR cells in the TG on day 7 following TNC or sham treatment. Arrows indicate FG-labeled TNFR2-IR cells. Scale bar: 100 μm. **b** Mean percentage of FG-labeled TNFR2-IR cells in the TG on day 7 following TNC or sham treatment. (*n* = 6 in each). **c** Effect of intra-TG etanercept or vehicle administration on day 4 on orofacial mechanical hypersensitivity following TNC or sham treatment. Data are presented as median, interquartile range (25–75 %), and maximum and minimum values. * *p* < 0.05. (*n* = 4–6; Kruskal-Wallis followed by Dunn’s multiple comparisons test)

TNFR2 expression was identified in FG-labeled TG neurons on day 7 after TNC or sham treatment, and many FG-labeled TG cells showed TNFR2-IR in TNC and sham rats (Fig. 3a). There was no significant difference in the number of TNFR2-IR TG neurons that innervate the whisker pad skin in the TNC and sham groups (Fig. 3b). Intra-TG administration of etanercept on day 4 after sham treatment did not change the MHWT of the ipsilateral whisker pad skin during the experimental period. TNC treatment decreased the MHWT of the ipsilateral whisker pad skin from day 3 to day 18, and the decrease in MHWT on day 7 was significantly suppressed by intra-TG administration of etanercept on day 4 after TNC (median on day 7, TNC + etanercept: 26 g, *n* = 7; TNC + vehicle: 10 g, *n* = 6; sham + Etanercept: 60 g, *n* = 5) (*p* < 0.05, Fig. 3c).

### P2X_3_R expression and its signaling in the TG following TNC

**Fig. 4:**
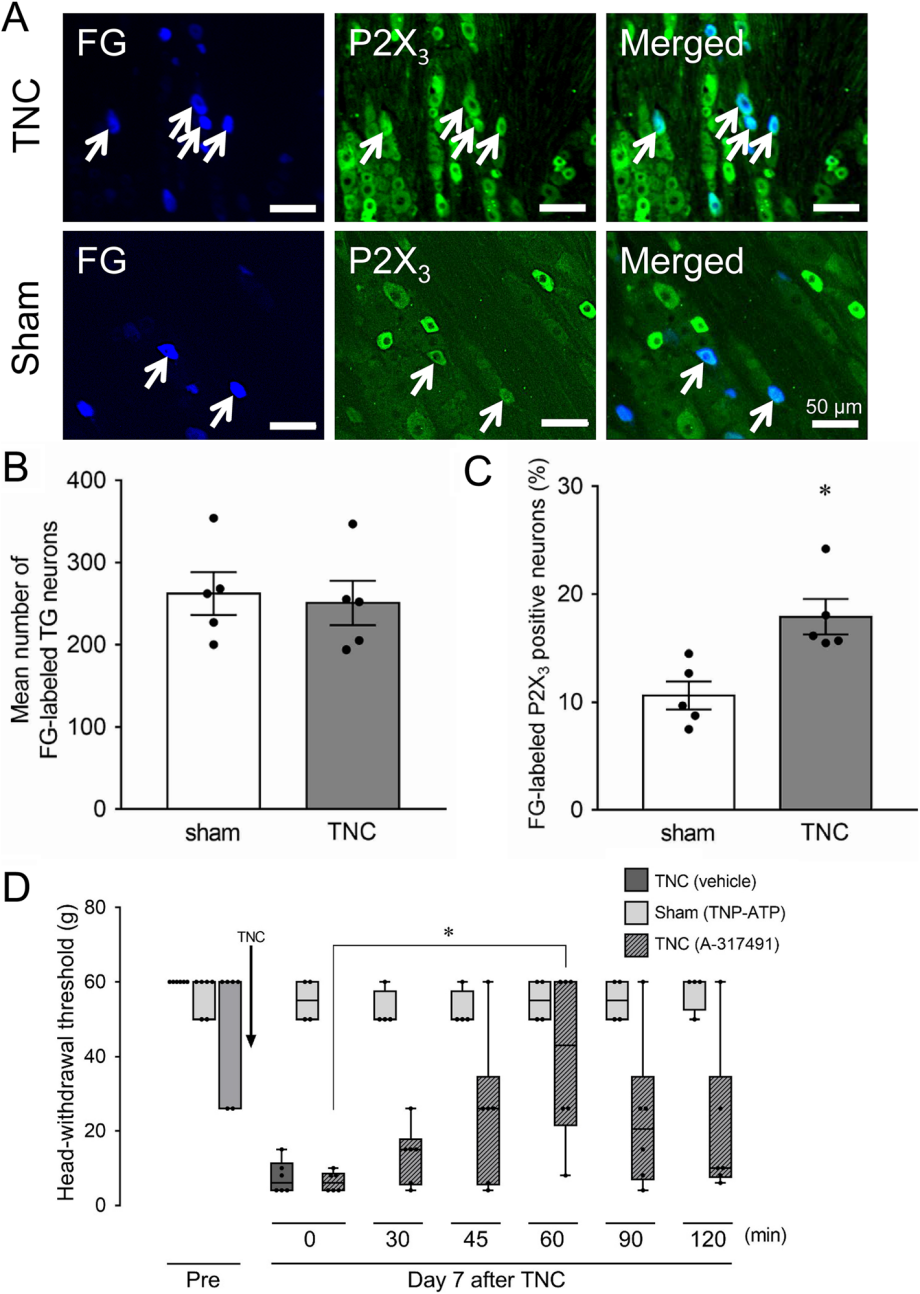
Changes in P2X_3_R expression in TG neurons innervating whisker pad skin and the involvement of P2X_3_R signaling in TG neurons in orofacial mechanical hypersensitivity on day 7 following TNC. **a** Photomicrograph of FG-labeled P2X_3_IR TG neurons following the TNC or sham procedure. Arrows indicate FG-labeled P2X_3_IR TG neurons. Scale bar: 100 μm. **b** Mean number of FG-labeled TG neurons ipsilateral to the TNC or sham procedure (*n* = 5 in each). **c** The frequency of FG-labeled P2X_3_-IR TG neurons ipsilateral to the TNC or sham procedure. * *p* < 0.05 vs. sham. (*n* = 5 in each, student’s *t*-test). **d** The time course of changes in the MHWT by subcutaneous A317491 or TNP-ATP administration on day 7 following the TNC or sham procedure. Data are presented as median, interquartile range (25–75 %), and maximum and minimum values. * *p* < 0.05. (*n* = 4–6; Kruskal-Wallis followed by Dunn’s multiple comparisons test)

P2X_3_R expression was identified in FG-labeled TG neurons on day 7 after TNC or sham treatment (Fig. 4a). Many FG-labeled cells were P2X3-IR in TNC rats, whereas a small number of them were P2X3-IR in sham-treated rats. TNC did not change the number of FG-labeled TG neurons compared with that of sham rats (TNC: 250.6 ± 27.0; sham: 262.0 ± 26.0, *n* = 6 in each) (Fig. 4b). On the other hand, the number of P2X_3_R-IR TG neurons that innervate the whisker pad skin was significantly higher in TNC (18 ± 1.6 %, *n* = 5) the sham group (11 ± 1.6, *n* = 5) (*p* < 0.05, Fig. 4c). TNC treatment decreased the MHWT of the ipsilateral whisker pad skin on day 7, and subcutaneous A-317491 administration into the whisker pad skin significantly recovered the decreased MHWT 60 min after the administration (median, 0 min: 6 g; 60 min: 43 g, *p* < 0.05, *n* = 6) (Fig. 4d). Subcutaneous TNP-ATP administration into the whisker pad skin did not change the MHWT after administration in the sham group.

### Effect of Etanercept administration on P2X_3_R expression

**Fig. 5:**
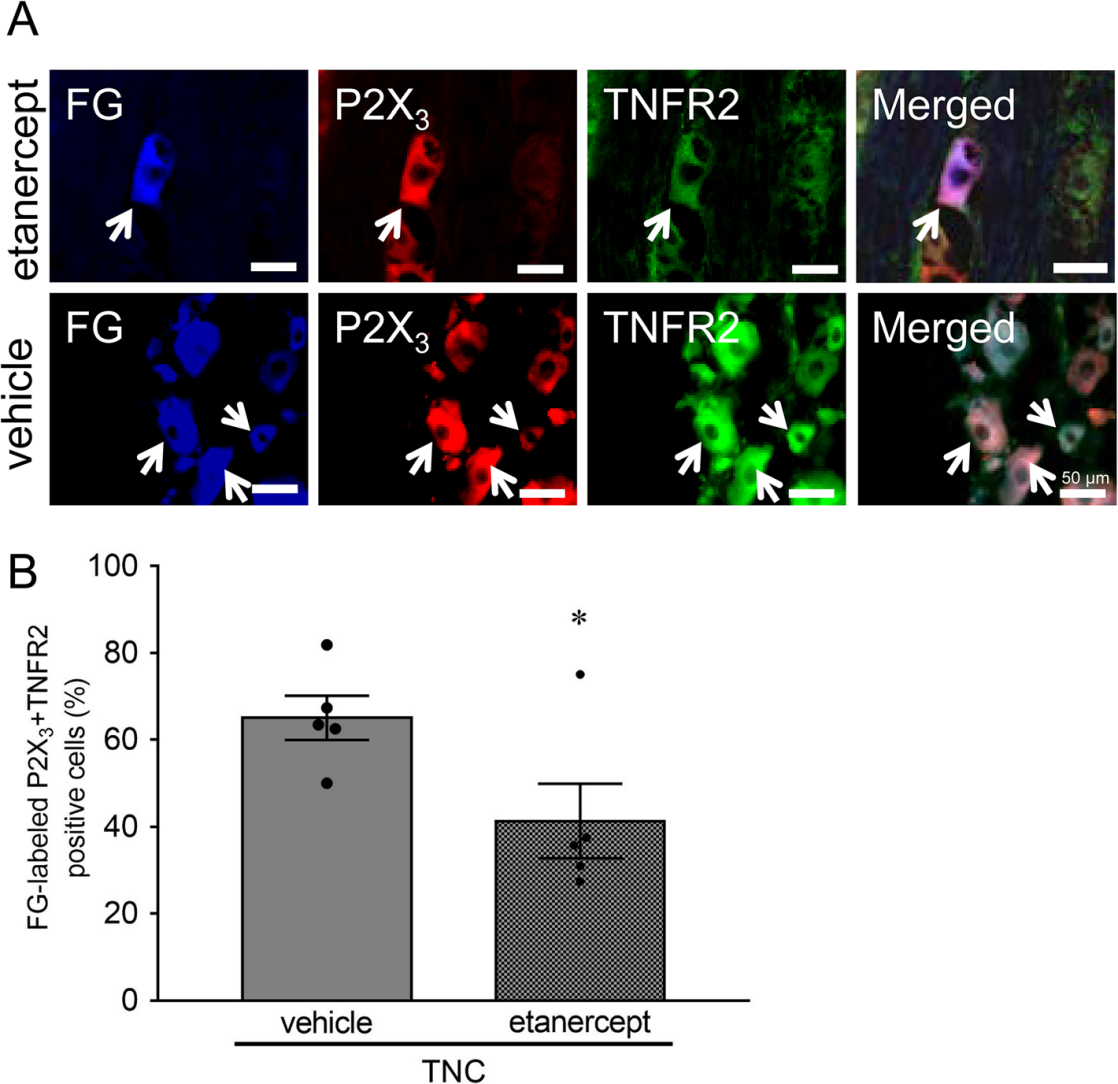
Involvement of TNFR2 signaling in the enhanced P2X_3_R expression in TG neurons on day 7 following TNC. **a** Photomicrograph of FG-labeled P2X_3_-IR and TNFR2-IR TG neurons following TNC with intra-TG etanercept or vehicle administration. Arrows indicate FG-labeled P2X_3_-IR and TNFR2-IR TG neurons. Scale bar: 100 μm. **b** Changes in the mean percentage of FG-labeled P2X_3_-IR and TNFR2-IR TG neurons on day 7 following TNC with intra-TG etanercept or vehicle administration. * *p* < 0.05 vs. vehicle. (*n* = 6 in each, student’s *t*-test)

P2X_3_R expression was identified in FG-labeled TNFR2-IR TG neurons after intra-TG etanercept and vehicle administration in the TNC group (Fig. 5a). Intra-TG etanercept administration significantly decreased the percentage of P2X_3_R-IR neurons in FG-labeled TNFR2-IR TG neurons ipsilateral to TNC (41.3 ± 3.8 %, *n* = 5) compared to that of intra-TG vehicle administration (65.0 ± 5.1 %, *n* = 5) (*p* = 0.05, Fig. 5b).

## Discussion

Clinically, TN can be classified into three types (classical, secondary, idiopathic) according to the diagnostic criteria issued by the international association for the study of pain [7]. Of these three types, unilateral orofacial neuropathic pain, which is caused by neuropathological changes in the trigeminal nerve root attributed to unilateral vascular compression and triggered by innocuous orofacial stimulation, is defined as the classical TN [18]. Typical patients with classical TN do not suffer from only orofacial neuropathic pain, which is typified by paroxysmal stabbing pain in the trigeminal territory, but also sustained orofacial pain [19]. At present, the first line of treatment for TN is oral administration of CBZ, which can stabilize a hypersensitized neural membrane by blocking the voltage-gated sodium channel, which is known to be effective in virtually all TN patients [20]. In this study, TNC induced unilateral transient orofacial mechanical hypersensitivity that mimics pain hypersensitivity in the trigeminal territory in TN patients, which was depressed by CBZ administration. These findings indicate that the TNC model sheds light on the mechanisms underlying orofacial neuropathic pain referred from TN. However, not all cases of TN present neurovascular contact on the symptomatic side, though CBZ treatment was confirmed to be effective in almost all TN patients [20, 21]. Moreover, about half of the TN patients are afflicted with concomitant spontaneous pain, which is rarely seen in the TNC model [18]. In addition, the TNC is not pulsating but permanent [18], which is different from vascular compression frequently underlying TN patients. Therefore, it is conceivable that the developed TNC model reflects a part of the neuropathophysiological condition of TN. In the future, it will be necessary to develop an animal model that is closer to the pathophysiology of TN.

Iba1 is notable as a specific marker of macrophages [22]. Moreover, the morphological features of macrophages change with an amoeboid-like appearance, indicating that their activation accumulates in the inflamed tissue [23]. Previous studies have indicated that peripheral nerve injury is conducive to increase in proliferated and/or resident macrophages in the TG or DRG [2, 24–26]. This is the first report that TNC, aside from peripheral nerve injury, induces the accumulation of macrophages showing amoeboid-like morphological changes into the TG, though a marked increase in OX42 (a marker of macrophages) immunoreactive cells was reportedly induced in non-neuronal structures in the spinal trigeminal nucleus in a TNC mouse model [27]. Previous studies have shown that the chemokine C-C motif ligand 2 (CCL2) released from damaged neurons, also designated as monocyte chemoattractant protein 1, and its signaling activates macrophages via the C-C chemokine receptor type 2 (CCR2), which elicits activation and proliferation of macrophages in the sensory ganglion [28, 29]. Additionally, peripheral nerve damage induces the enhancement of CCL2 expression and macrophage accumulation in sensory neurons through toll-like receptor 2 (TLR2) signaling [30]. From these studies, it can be assumed that TNC facilitates the remarkable accumulation of activated macrophages in the TG, which is likely induced by CCL2 signaling through TLR2 activation. However, it is not yet known whether exogenous macrophages infiltrate into the TG or activated macrophages proliferate in the TG; further research is needed to determine the origin of the increased macrophages in the TG.

Following peripheral nerve injury activated macrophages in the sensory ganglion release TNFα [31, 32], whose levels are reported to be increased as well [33, 34]. TNF-α release associated with macrophage activation accelerates intracellular signal transduction pathways, including extracellular signal-regulated kinase (ERK) and p38 mitogen-activated protein kinase (MAPK) cascades [35]. Peripheral nerve injury enhances the production of substance P (SP), which is a well-known mediator of neuroimmunomodulation and released from DRG neurons [36]. SP signaling regulates not only various functions of macrophages but also accelerates ERK1/2 and p38 MAPK cascades in macrophages [37]. Furthermore, the facilitation of the p38 MAPK cascade in macrophages infiltrates the nerve-injured region, resulting in TNFα production from macrophages [38]. Based on the present results, the accumulation of activated macrophages that express TNFα in the TG was immunohistochemically confirmed. TNC remarkably increased macrophage and TNFα protein expression in the ipsilateral TGs according to TNC. Taken together, these results suggest that the signaling of SP, which was likely released from TG neurons associated with TNC, activates macrophages in the TG, resulting in TNFα release from activated macrophages via the ERK1/2 and p38 MAPK signaling cascades.

In this study, P2X_3_R expression was identified in TNFR2-positive TG neurons innervating the whisker pad skin following TNC. The number of P2X_3_R-IR TG neurons that innervate the whisker pad skin was significantly increased following TNC. TNFα release from activated macrophages occurred in the TG following TNC, and the TNFR2 antagonism in the TG significantly diminished the TNC-induced increase of P2X_3_R-IR TG neurons innervating the whisker pad skin. Moreover, subcutaneous P2X_3_R antagonism in the whisker pad skin significantly depressed TNC-induced mechanical hypersensitivity. P2X_3_R, which is a ligand-gated ion channel, is expressed in small-diameter TG neurons; the upregulation of P2X_3_R-positive TG neurons after trigeminal nerve injury reportedly leads to trigeminal neuropathic pain [39]. It has been reported that exogenous TNFα by intra-DRG administration elicits DRG neuronal depolarization and hyperexcitability [40]. TNFα signaling is known to induce ERK phosphorylation in cultured DRG neurons [41], and increased ERK signaling upregulates the transcription of the P2X_3_R gene [42]. Together, these results suggest that TNC-induced orofacial mechanical hypersensitivity is, at least in part, dependent on the upregulation of P2X_3_R expression in TG neurons innervating the orofacial region via the increased TNFR2/ERK signaling cascade. In addition, trigeminal nerve injury activates satellite glial cells through the TG via gap junctions [1]; TNFα is released from activated satellite glial cells through enhanced P2X_7_ signaling following peripheral sensory nerve injury [43, 44].

Alternatively, following peripheral nerve injury, the voltage-gated sodium channel activation is augmented by TNFα signaling in DRG neurons in a concentration-dependent manner depending on the upregulation of tetrodotoxin (TTX)-sensitive and resistant current densities via the increased intracellular p38 MAPK signaling [34, 45, 46]. Membrane potassium ion conductance is also increased by TNFα signaling in a non-voltage-gated fashion, resulting in sensory neuronal hyperexcitability [47, 48]. These mechanisms to enhance sensory neuronal excitability by TNFα signaling are also potentially involved in TNC-induced orofacial mechanical hypersensitivity. However, the detailed mechanisms remain unknown. Therefore, further studies are required.

## Conclusions

In conclusion, TNC could induce unilateral continuous orofacial mechanical hypersensitivity that mimics pain hypersensitivity in the trigeminal territory in patients with TN. The signaling of TNFα released from the activated macrophages and infiltrated into and/or proliferated in the TG induces the upregulation of P2X_3_R expression in TG neurons innervating the orofacial region, resulting in orofacial mechanical allodynia following TNC. Consequently, TG neuronal P2X_3_R hyperexpression by enhanced TNFα signaling in the TG is a potential target for TN treatment.

## Data Availability

The raw data supporting the conclusions of this article will be made available by the authors on request, without undue reservation.

## Abbreviations

P2X3R: P2X_3_ receptor
TG: Trigeminal ganglion
TNFα: Tumor necrosis factor alpha
TNC: Trigeminal nerve root compression
TNFR2: TNF receptor-2
TN: Trigeminal neuralgia
DRG: Dorsal root ganglion
MHWT: Mechanical head-withdrawal threshold
FG: FluoroGold
PB: Phosphate buffer
PBS: Phosphate buffered saline
Iba1: Ionized calcium binding adapter protein 1
BSA: Bovine serum albumin
CBZ: Carbamazepine
ANOVA: Analysis of variance
CCL2: Chemokine C-C motif ligand 2
TLR2: Toll-like receptor 2
ERK: Extracellular signal-regulated kinase
MAPK: Mitogen-activated protein kinase
SP: Substance P
TTX: Tetrodotoxin
